# Prevalence of metabolic syndrome and associated factors among psychiatric patients at University of Gondar Comprehensive Specialized Hospital, Northwest Ethiopia

**DOI:** 10.1371/journal.pone.0256195

**Published:** 2021-08-26

**Authors:** Nigist Alemayehu Woldekidan, Ammas Siraj Mohammed, Amsalu Degu, Yohannes Tadiwos

**Affiliations:** 1 Department of Clinical Pharmacy, School of Pharmacy, College of Medicine and Health Sciences, University of Gondar, Gondar, Ethiopia; 2 Department of Clinical Pharmacy, School of Pharmacy, College of Health and Medical Sciences, Haramaya University, Harar, Ethiopia; 3 School of Pharmacy and Health Sciences, United States International University Africa, Nairobi, Kenya; 4 Department of Pharmacology, School of Pharmacy, College of Health and Medical Sciences, Haramaya University, Harar, Ethiopia; Università degli Studi di Milano, ITALY

## Abstract

**Background:**

Psychiatric patients are at increased risk of being overweight or obese, and subsequently develop metabolic syndrome. Nevertheless, data regarding associated factors for weight gain are limited and inconsistent.

**Objective:**

The present study aimed to determine the risk of metabolic syndrome and its associated factors among psychiatric patients.

**Method:**

A cross-sectional quantitative study was conducted among all psychiatric patients at the Psychiatric Unit of the University of Gondar Comprehensive Specialized Hospital from March 1- April 1, 2018. All eligible psychiatric patients were interviewed about their socio-demographic status,and clinical characteristics and useful parameters for the study were recorded from the medical records of the patients and by measuring waist to height ratio. Descriptive statistics were used to summarize baseline information.Binary logistic regression was used to determine the associated factors and P-value <0.05 and confidence interval (CI) of 95% were used as cut off points for determining statistical significance.

**Result:**

From 300 patients included in the study, 168(56%) patients were females,and around 50.3% of the study participants had low literacy levels. As per waist to a height ratio scale, 58% (174) of the patients had a risk of metabolic syndrome. The Binary logistic regression analysis indicated that sex (p-<0.0001), occupation (p -0.032), marital status (p-0.006), and distance from the hospital (p<0.0001) were statistically significant determinants of metabolic syndrome risk in the psychiatric patient in our setting.

**Conclusion:**

The majority of the psychiatric patients in the study setting had a risk of metabolic syndrome. Sex, marital status, employment status, and distance to the hospital were significantly associated with metabolic syndrome. Routine physical and laboratory investigations to detect metabolic syndrome are indispensable in psychiatric patients to prevent cardiovascular complications.

## Background

Metabolic Syndrome (Mets) is a worldwide health problem that can be defined as a constellation of risk factors including glucose intolerance, raised blood pressure, obesity, and dyslipidemia, which increases cardiovascular morbidity and mortality rate [1]. During the last two decades, the frequency of obesity and diabetes mellitus has increased in the United States, which is directly associated with cardiovascular disease (CVD) [2] When compared to people without MetS, the chance of heart attack or stroke is three times more and the chance of death because of cardiovascular complication is twice higher in MetS patients [3].

In contrast to the general population, patients with schizophrenia have a higher rate of Mets [4]. As a result of this, these patients are at increased risk of premature CVD and subsequent shorter life expectancy [5–7]. A previous study showed that 31–34% of the population with severe and persistent psychiatric disorders complicated by Metsdied from CVD, which indicates how much Mets directly increases the risk of developing CVD [8].

Abnormally large waist circumference (WC) has been debated in the literature as a controversial measurement parameter for the assessment of metabolic Mets. It was not consistently considered as a diagnostic criterion for determining Mets [9]. However, increased WC is the main feature of Mets, and it is associated with high fasting blood glucose (FBG) and hypertension as well as lipid dysregulation. Other metabolic risk factors include central obesity and insulin resistance [10,11]. The waist height ratio (WhtR) has been recommended as a simple cost, and noninvasive screening tool for psychiatric patients receiving antipsychotic medications to assist in identifying metabolic screening [12]. Although Mets in psychiatric patients is a burning issue causing CVD, the screening guidelines are still not widely applied, even in developed countries [13,14]. Besides, the studies conducted regarding Mets risk show inconsistent results. Therefore, the present study was aimed to determine the risk of Mets and its associated factors among psychiatric patients in the study setting, since limited data is available in the current study area regarding metabolic risk and associated factors.

## Methods

### Study design, setting and period

A cross-sectional study was conducted among inpatient and outpatient psychiatric patients attending the University of Gondar Comprehensive Specialized Hospital (UOGCSH). UOGCSH is one of the oldest teaching hospitals in the country, located in Gondar town, and it is 738km northwest of the capital city Addis Ababa, Ethiopia. The study was conducted from March 1- April- 30, 2018.

### Inclusion and exclusion criteria

All patients aged 18 years and above who received treatment for at least six months from the inpatient or outpatient psychiatry clinic and who attended the clinic during the study period were included in the study. Nonetheless, unstable psychiatric patients were excluded from the study due to the inability to interview and measure the required metabolic parameters of the patients.

### Sample size determination

All eligible psychiatric patients who fulfilled the inclusion criteria and who received treatment in the inpatient and outpatient Psychiatric departments during the study period were included as the study sample.

### Study variables

The independent variable includes age, gender, religion, ethnicity, education status, occupation, marital status, area of residence, distance from the hospital, and monthly income. In contrast, the dependent variable is the Mets risk.

### Data collection and management

The data collection was performed by two well-trained psychiatric nurses who were working at the psychiatry clinic in UOGCSH, and, who have professional experience in approaching and drawing necessary information from psychiatric patients.Necessary training was provided by pricipal investigator to data collectors on contents of the questionnaire, the data collection methods and ethical concerns. The data was collected through face-to-face interviews and hospital files of the patients using data collection instruments and measuring essential parameters from the patients. The data collection instrument was adapted from the previous studies [12,15,16], and it includes sections focusing on socio-demographic, other baseline characteristics, and clinical characteristics. WhtR was calculated by dividing waist circumference (in inches) by height (in inches). Waist circumference was measured midway between the inferior costal margin and the superior border of the iliac crest at the end of normal expiration in a standing position. While height was determined using the wall-mounted measuring tape in the ward, and the participants were not wearing shoes. The WhtR parameter of over 0.50 was considered at risk ofMets [12].

### Data quality assurance

The data collection instrument was also pretested on fifteen patients who were not included in the final analysis, and then it was validated and pertinent modifications were made before the beginning of actual data collection. Data collectors were trained well on the measurement of waist circumference and height to make the result consistent throughout the collectors. The investigators were also trained on ways of approaching the patients and upkeep their permission for an interview before the data collection process.

### Data analysis

The collected data were entered and analyzed using Statistical packages for Social Sciences (SPSS) version 20 statistical software. Frequencies and percentages were used to describe socio-demographic and other characteristics, and binary logistic regression was done to determine the association among variables. P-value <0.05 and 95% confidence interval (CI) were used as cut points for determining statistical significance.

### Ethical consideration

Ethical clearance was obtained from the Research and Ethics Review Committee, School of Pharmacy, University of Gondar. Then, permission to collect data from patients was obtained from the Clinical Directorate of UOGCSH. Names and other personal identifications were eliminated during data collection to ensure the confidentiality of the information collected. Written informed consent and oral explanations about the purpose of the study were provided to all the participants during data collection and participation was voluntary based. The capacity to participate was determined by asking the participants understanding of the purpose of the study.

## Result

A total of 300 patients were interviewed, of which 168 (56%) were females, and most of them 145 (48.3%) were in the age group 18–30 years (Mean age 31.97). Half (151,50.3%) of the participants had low literacy levels and were self-employed (156, 52%). Nearly half of them live in rural areas 167(55.7%) and more than two-third of participants are from ≤ 40km distance (220, 73.3%).

The present study showed that schizophrenia (147, 49%) was the most commonly diagnosed psychiatric disorder in the study setting. Major depression (100, 33.3%) was the second leading case diagnosed in this setting. On the other hand, brief psychosis and generalized anxiety disorders were the least common psychiatric disorders in our setting. The majority of the study participants (138, 46%) had ≤ five years of duration of illness, and 95% of the patients had been treated with pharmacotherapy for a period of fewer or equal to ten years. Besides, haloperidol was the most commonly prescribed antipsychotic in the study setting (Table 1). As per waist to a height ratio scale, 58% (174) of the patients had a risk of metabolic syndrome.

**Table 1:** Socio-demographic characteristics and clinical features of participants.

Variable	Category	N	Percentage %
**Age**	18–30	145	48.3
	31–40	100	33.3
	41–50	38	12.7
	51–60	17	5.7
**Sex**	Male	132	44
	Female	168	56
**Religion**	Orthodox	237	79
	Muslim	67	20.3
	Protestant	2	7
**Ethnicity**	Amhara	293	97.7
	Tigray	7	2.3
**Education Status**	Unable to read and write	151	50.3
	Primary school (1–8)	56	18.7
	Secondary school (9–10)	56	18.7
	Tertiary school	37	12.3
**Occupation**	Government employed	16	5.3
	Self- employed	156	52
	Unemployed	128	42.7
**Marital Status**	Single	107	35.7
	Married	193	64.3
**Area of Residence**	Urban	133	44.3
	Rural	167	55.7
**Monthly income in ETB**	<500	214	71.3
	500–1499	66	22
	1500–2499	11	3.7
	>2500	9	3
**Distance from the hospital (in km)**	≤40km	220	73.3
	>40	80	26.7
**Type of mental illness**	Schizophrenia	147	49
	Major depression	100	33.3
	Bipolar disorder	26	8.7
	Generalized Anxiety	13	4.3
	Brief Psychosis	14	4.7
**Duration of Illness**	<5 year	138	46
	6–10 year	116	38.7
	11–15 year	30	10
	16–20 year	7	2.3
	>21 years	9	3
**Years of Pharmacotherapy**	≤ 10years	285	95
	>10 years	15	5.0
**Drug prescribed**	Haloperidol	92	30.7
	Chlorpromazine	79	26.3
	Chlorpromazine with amitriptyline	25	8.3
	Fluoxetine	55	18.3
	Carbamazepine	7	2.3
	Risperidone	15	5
	Amitriptyline	27	9
**Medication Cost/day (ETB)**	≤5	291	97
	>5	9	3

In the binary logistic regression analysis, the study indicated that male patients had an 88% decreased risk of Metsas compared to female patients (AOR = 0.191, 95% CI = 0.093–0.391, p- <0.0001). Besides, participants who were government workers (AOR = 1.009, 95% CI = 0.168–6.052, p = 0.032) and self-employed (AOR = 0.339, 95% CI = 0.143–0.802, p = 0.032) were 1.009 and 0.339 times less likely to develop risk of Metsas compared to unemployed patients, respectively. The current study also showed that being single had a 73% decreased risk of acquiring Metsin contrast to married patients (AOR = 0.270, 95% CI = 0.107–0.684, p = 0.006). On the other hand, patients who were living closer to the hospital had a higher chance of getting Mets. However, the other parameters did not show a statistically significant association with the Mets risk (Table 2).

**Table 2:** Univariable and multivariable binary logistic regression analysis of predictors of metabolic syndrome risk.

Variable	Category	COR (95%CI)	AOR (95%CI)	p-value
Age	18–30	0.682[0.228–2.040]	1.315[0.188–9.169]	0.984
	31–40	0.741[0.242–2.271]	1.128[0.171–7.422]	
	41–50	0.639[0.187–2.185]	1.114[0.151–8. 213]	
	51–60	1	1	
Sex	Male	0.207[0.127–0.343]	0.191[0.093–0.391]	<0.0001**
	Female	1	1	
Ethnicity	Amhara	1.285[0.282–5.849]	0.472[0.061–3.628]	0.471
	Tigray	1	1	
Education Status	Unable to read and write	1.033[0.492–2.168]	2.350[0.655–8.433]	0.325
	Primary School (1–8)	1.522[0.630–3.675]	2.759[0.665–11.448]	
	Secondary School (9–10)	0.755[0.323–1.762]	1.225[0.352–4.260]	
	Tertiary School	1	1	
Occupation	Government employed	0.377[0.129–1.099]	1.009[0.168–6.052]	0.032*
	Self- employed	0.316[0.188–0.532]	0.339[0.143–0.802]	
	Unemployed	1	1	
Marital Status	Single	0.633[0.390–1.029]	0.270[0.107–0.684]	0.006*
	Married	1	1	
Area of Residence	Urban	1,438[0.892–2.316]	0.696[0.335–1.445]	0.331
	Rural	1	1	
Monthly income in ETB	<500	2.627[0.684–10.089]	1.540[0.199–11.947]	0.269
	500–1499	1.411[0.348–5.728]	0.596[0.083–4.280]	
	1500–2499	1.042[0.177–6.123]	1.267[0.089–17.942]	
	>2500	1	1	
Distance to the hospital(in km)	≤40km	3.000[1.770–5.084]	4.530[2.002–10.252]	<0.001**
	>40km		1	
Type of mental illness	Schizophrenia	0.188[0.041–0.871]	0.737[0.103–5.273]	0.196
	Major depression	0.500[0.105–2.389]	3.536[0.154–81.167]	
	Bipolar disorder	0.267[0.049–1.449]	3.203[0.298–34.363]	
	Generalized Anxiety	0.267[0.041–1.727]	3.705[0.105–130.891]	
	Brief Psychosis	1	1	
Duration of Illness	<5 year	0.458[0.092–2.289]	1.196[0.051–28.266]	0.609
	6–10 year	0.564[0.112–2.845]	1.071[0.049–23.436]	
	11–15 year	0.374[0.066–2.106]	0.723[0.036–14.469]	
	15–20 year	0.214[0.024–1.877]	0.147[0.005–4.234]	
	>21 years	1	1	
Years of Pharmacotherapy	≤ 10 years	0.844[0.281–2.537]	0.323[0.030–3.422]	
	>10 years	1	1	
Drug indicated	Haloperidol	0.159[0.051–0.491]	0.212[0.014–3.320]	0.099
	Chlorpromazine	0.376[0.117–1.202]	0.625[0.039–10.051]	
	Chlorpromazine with amitriptyline	0.913[0.202–4.120]	0.740[0.113–4.858]	
	Fluoxetine	0.304[0.092–1.006]	0.338[0.066–1.734]	
	Carbamazepine	0.130[0.021–0.817]	0.070[0.002–2.149]	
	Risperidone	0.261[0.059–1.148]	0.088[0.002–4. 162]	
	Amitriptyline	1	1	
Medication Cost/day	≤5	0.847[0.208–3.457]	0.355[0.017–7.287]	0.501
	>5	1	1	

*Significant at 0.05 levels

**significant at 0.01 levels.

COR: Crude odds ratio, AOR: Adjusted odds ratio, CI: Confidence interval, ETB: Ethiopian Birr.

## Discussion

Based on WhtR value over 0.50 as a parameter that reveals confirmation of abdominal obesity, 58% of participants had abdominal obesity, which directly indicates Metsrisk. One factor that could lead to such increased metabolic risk is the duration of the illness, with more than half (54%) of the patients having a duration of illness greater than 5 years. There is some eviedence for a dose-responce association with the severity and duration of symptoms and for a bidirectional longitudinal impact between psychatric disorders and Mets. Associations generally seem stronger with abdominal obesity and dyslipidemia dysregulation than with hypertension. Contributing mechanisms are an unhealthy life style and a poor adherence to medical regimen which are prevalent among psychiatric patients. Specific psychiatric medications have also shown a profound impact in increasing Mets dysregulations. Finally, pleiotropy in genetic vulnerablity and pathophysiological mechanisms, such as those leading to the increased central and peripherial activation of immunometabolic or endocrine symptoms, plays in both Mets and psychiatric disorder development [17].

Previous studies showed that the risk of Mets was prevalent among patients on second-generation antipsychotics [18,19]. However, in our setting due to the limited availability of second-generation antipsychotics, first-generation antipsychotics such as haloperidol was frequently prescribed. Hence, the lack of a significant association between Mets risk and the prescribed medications in our setting could be probably due to the prevalent use of first-generation antipsychotics which has a lower risk of Mets. Related to the specific case, it has been reported that the prevalence of Mets in patients with major depression ranges from 36–50% [16]. Nevertheless, there was no significant association identified between Mets and the types of psychiatric disorders in our setting. The finding of the study indicated that females had an increased risk than males which is in line with a study conducted in South Africa [20] and Brazil [21]. the study done in jimma University Specialized Hospital, Southeast Ethiopia also showed a significant association of Mets with being female [22]. Another study also showed that the prevalence rate of Metswas 51.6–54.2% in women and 36–36.6% in male [23]. This increased risk in women might be because of difference in body composition, with women having more fat mass. Different conditions such as pregnancy, oral contraceptive therapy, and menopause could also contribute to Mets [24].

Significant association between employment status and Metsrisk was shown in our study. A previous study showed that there is a relationship between employment status and physical inactivity, which was explained mainly by health-related and socioeconomic factors [25]. Another study also revealed that there was an association between physical inactivity and Mets [26]. A significant association in the current study could also be because of the higher unemployment, which leads to stress imposes peoples to be stressed and stress is thought to influence eating behavior. Stress is more likely to increase food intake, along with high consumption of palatable food, rich in saturated fat and sugar [27].

Our study also revealed that those who were single have an increased risk for Mets than married/ever married. Similarly, a study conducted in this area showed that marital status to be of important factor. Here, living single was to be associated with a poorer lifestyle and higher CVD risk, at least in males [28]. Another study also reported marital quality as one of the important factors that mediate the association between marital status and Mets [29].

Furthermore, participants who were relatively near to the hospital were at risk of Mets than those far away. The possible reason could be as the hospital is in an urban area, the majority of the study population (73.3%) are living near the hospital, having a sedentary life and eating an unhealthy diet. However, an insignificant association was found between living in the urban area (44.3% of the patients) and Mets. This could be because even though the patients are residing in the rural areas, the distance from their home to the hospital might be lesser than 40 km as is evident by the result which shows that 73.3% of the participants are living near the hospital at a distance of less than 40 km.

Psychiatric patients are at increased risk of Mets and this risk is present for a range of psychiatric conditions, including major depressive disorder, bipolar disorder, schizophrenia, anxiety disorder, attention-deficit/hyperactivity disorder, and posttraumatic stress disorder [17]. In the current study, no correlation was found between the type of psychiatric disorder and the risk of Mets. One possible reason might be the prolonged duration of the mental disorder that is observed in the majority of the study participants (54%), increasing the chance of metabolic disorder irrespective of the type of the type of psychiatric disorder.

## Limitation of the study

Identifying patients with Mets and other comorbid conditions with the help of laboratory parameters in the psychiatric care unit was not carried out in our setting. For that reason, the study was unable to differentiate patients who already have Mets. In addition to this, as it is a cross-sectional study, confounding variables cannot be controlled to identify definitive exposing factors for Mets at risk. The lack of information regarding patients’ awareness of suffering from clinical conditions associated with metabolic syndrome could also be considered as a possible limitation since adequate awareness of individual cardiovascular risk profile is a key element for improving adherence to life-style rules and adoption of preventive treatment strategies [30].

## Conclusion

The present study showed that long-term psychiatric patients are at increased risk of Mets. Sex, marital status, employment status, and distance to the hospital were statistically associated with Mets. Therefore, routine physical and laboratory investigations to detect Mets are indispensable to prevent cardiovascular complications.

## Supporting information

S1 File Metabolic syndrome among psychiatric out patients. (PDF)

## Abbreviations

AOR: Adjusted Odd Ratio
COR: Crude Odd Ratio
CVD: Cardiovascular Disease
ETB: Ethiopian Birr
FBG: Fasting Blood Glucose
Mets: Metabolic Syndrome
UOGCSH: University of Gondar Comprehensive Specialized Hospital
WC: Waist Circumference
WhtR: Waist per height ratio
