# Supplementary material for: Prevalence of metabolic syndrome and associated factors among psychiatric patients at University of Gondar Comprehensive Specialized Hospital, Northwest Ethiopia
Source: PLoS One. 2021 Aug 26;16(8):e0256195. doi: 10.1371/journal.pone.0256195 (PMC8389454; doi:10.1371/journal.pone.0256195)
Supplement: S1 File — (PDF) [file pone.0256195.s001.pdf]

Prevalence of Metabolic syndrome and associated factors among psychiatric patients at University of Gondar Comprehensive Specialized Hospital, Northwest Ethiopia

1. Age 1. 18-30 2.31-40 3.41-50 4.51-60
2. Sex: 1. Male 2. Female
3. Religion: 1. Orthodox 2. Muslim 3. Protestant 4. Others _______
4. Ethnicity: 1. Amhara 2. Tigre 3. Others___________
5. Education status: 1. Unable to read and write 2. Only able to read and write 3. Primary school (1-8)
   4. Secondary school (9-10) 5. Preparatory 6. College and above
6. Occupation: 1. Government employed 2. Self –employed 3. Unemployed 4. Student
7. Marital status: 1. Single 2. Married 3. Divorced 4. Widowed

8. Area of Residence: 1. Urban 2. Rural
9. Monthly income (Birr): 1. <500 2. 500- 1499 3. 1500-2499 4. >2500 5. No income
10. Distance from the hospital (in Km) 1. <20 2. 21-40 3. 41-60 4 . >60
11. Type of mental illness -----------------
12. Duration of illness 1. <5yr 2. 6-10yr 3. 11-15yr 4.16-20 yr 5. >21yr
13. Years of pharmacotherapy 1. <5yr 2. 6-10yr 3. 11-15yr 4.16-20 yr 5. >21yr
14. Drug (s) prescribed ----------------------------
14. Medication cost/day ------------------------------
15. Waist circumference (cm) ----------------------------
16. Height -------------------
